# Do Psychological Factors Affect Life Satisfaction and Pain Interference in Spine Surgery Patients? A 12-Month Follow-Up Study

**DOI:** 10.3390/jcm13237007

**Published:** 2024-11-21

**Authors:** Emma Lappalainen, Jukka Huttunen, Hannu Kokki, Petri Toroi, Merja Kokki

**Affiliations:** 1 Institute of Clinical Medicine, University of Eastern Finland, FI-70211 Kuopio, Finland; emmla@student.uef.fi (E.L.); hannu.j.kokki@gmail.com (H.K.); 2 Department of Neurosurgery, Neuro Centre, Kuopio University Hospital, FI-70029 Kuopio, Finland; jukka.huttunen@pshyvinvointialue.fi; 3 Department of Anaesthesiology and Intensive Care, Kuopio University Hospital, FI-70029 Kuopio, Finland; petri.toroi@pshyvinvointialue.fi

**Keywords:** pain, postoperative, oxycodone, tramadol, paracetamol, life satisfaction, resilience, anxiety, depression

## Abstract

**Background/Objectives**: Psychological factors impact patient-reported outcome measures (PROMs). This study assessed the influence of preoperative anxiety, depression, and resilience on postoperative pain interference and life satisfaction one year after spine surgery. **Methods**: This study was a secondary analysis of a study involving 120 patients who underwent elective spine surgery and were randomly assigned to receive either tramadol–paracetamol (37.5 mg/325 mg; two tablets; n = 61) or placebo (n = 59) twice per day for pain management during the first five postoperative days. Patients completed the Life Satisfaction Scale-4, Brief Pain Inventory, Hospital Anxiety and Depression Scale, and Resilience Scale-14 questionnaires before surgery and at 28 days and 52 weeks post surgery. The primary outcomes were life satisfaction and pain interference at 12 months after spine surgery and their associations with preoperative anxiety, depression, and resilience. **Results**: Data from 113 patients (94% response rate) were collected at 52 weeks postoperatively. The number of patients reporting satisfaction with their life increased from three (5%) and two (3%) before surgery to 23 (41%) and 19 (34%), while pain interference decreased from a median of 4.1 to 1.2 and from 4.4 to 1.9 on a scale of 0–10 at 12 months in the placebo and tramadol–paracetamol groups, respectively. The linear regression analysis revealed no statistically significant predictive value for preoperative anxiety, depression, or resilience score for life satisfaction and pain interference at 12 months after spine surgery. **Conclusions**: These results highlight that psychological factors, anxiety, depression, and resilience did not have an impact on postoperative pain outcomes and life satisfaction in patients undergoing spine surgery.

## 1. Introduction

The global burden of back pain is substantial, and back pain is the leading cause of disability [1,2]. Most patients with back pain can be treated conservatively, although some patients, such as those with persistent radicular symptoms, require surgical treatment aimed at providing effective pain relief and improved performance [3].

However, not all patients benefit from surgery, and some develop persistent spinal pain syndrome (PSPS). Patients with PSPS have chronic or recurrent pain, often with neuropathic pain features, such as paresthesia, numbness, and muscle weakness originating from the spine [4,5,6].

In person-centered and value-based care, patient-reported outcome measures (PROMs) are important assessment tools for evaluating surgical outcomes from the patient’s perspective [7]. Patients’ psychological characteristics can influence PROMs. Preoperative anxiety and depression are examples of psychosocial risk factors associated with increased postoperative pain [8,9,10]. Recently, we found that greater preoperative resilience, which refers to a person’s adaptive capacity in the face of adversity [11], is associated with less pain up to 12 months after surgery [12]. Supporting data regarding patients with spinal fusion were published by Daher et al., who found that low resilience before surgery is a predictor of poor postoperative outcomes [13].

Patients suffering from life dissatisfaction have more psychological distress [14]. Life dissatisfaction is important for patients’ health since it is associated with increased all-cause mortality [15]. In the spine surgery patient population, life dissatisfaction is common, but recently, we found that spine surgery may enhance life satisfaction in selected back patients. Moreover, pain interference is associated with life satisfaction in this patient population [16].

Preoperative anxiety, which is mainly related to surgical factors, is common in surgical patients of all ages, and thus, prehabilitation has been proposed [17]. Preoperative education could support patients’ psychological, economic, and clinical rehabilitation [18]. Positive affect during early recovery after surgery is associated with better functional outcomes, while negative affect is linked to increased pain-related disability and pain interference after surgery. Depression during the early phase of recovery is also associated with increased pain intensity, pain interference, and disability at 3 months after surgery [19]. However, neither positive nor negative affect nor depression seems to have the same associations when measured before surgery, which is why mental health should be evaluated and supported also postoperatively in spine surgery patients. Interventions, including positive psychology, affect pain perception and decrease pain intensity [20]. Positive psychology interventions support patients’ psychological well-being, positive resource capacity, and adaptation skills, and these methods could be usable in the spine surgery patient population, e.g., as a digital health intervention, which is feasible both in preoperative and postoperative care [21].

Tramadol is an analgesic with pain-relieving effects as a weak opioid, mainly through its metabolite O-desmethyltramadol. Tramadol also acts as a noradrenalin and serotonin reuptake inhibitor (SNRI) [22]. Due to its dual mechanism of action, tramadol affects both nociceptive and neurogenic pain [23]. Based on its SNRI action, it also relieves anxiety and depressive symptoms [24]. Tramadol–paracetamol combination products have proven analgesic efficacy in various patient groups, including those who undergo orthopedic surgery and those with low-back pain [25,26]. This combination has a supra-additive effect in pain relief, and it prevents hyperalgesia [27,28]. However, in our previous publication on this patient cohort, tramadol–paracetamol combination products used as add-on treatments were not superior to placebo for acute pain management after spine surgery [29].

This secondary analysis of a randomized placebo-controlled double-blinded clinical trial [29] aims to evaluate PROMs, i.e., life satisfaction, as well as pain interference after spine surgery and the effects of patients’ psychological factors, including anxiety, depression, and resilience on these two PROMs. The effects of tramadol–paracetamol for early postoperative pain management on PROMs and psychosocial factors were also evaluated.

## 2. Materials and Methods

This is a large study, and some results regarding pain recovery have been published previously [29].

### 2.1. Study Design and Participants

**Figure 1:**
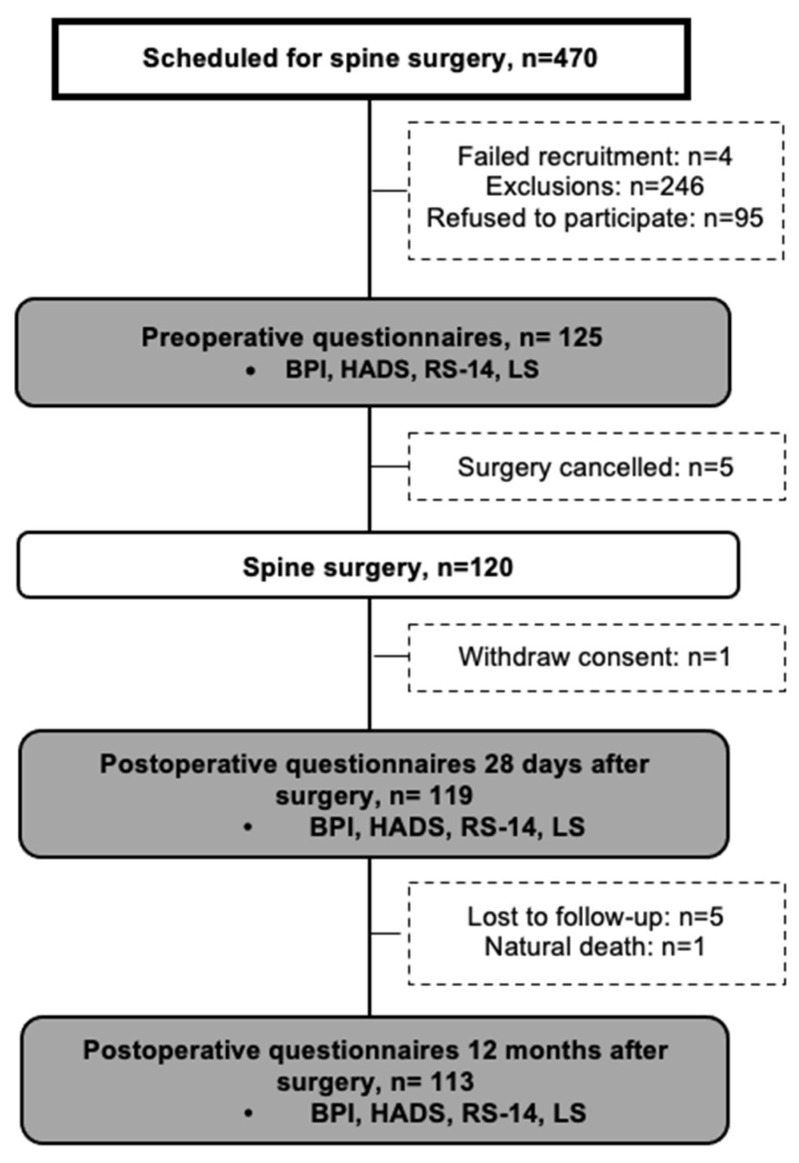
Flow chart.

Between September 2019 and February 2021, 120 patients were enrolled in this prospective, randomized, placebo-controlled, double-blind study. Patients aged 18–75 years with a body mass index of 18–35 kg/m^2^ who were scheduled to undergo lumbar or cervical spine surgery at Kuopio University Hospital were included. Patients were excluded if they had contraindications for the study compounds or a history of alcohol or drug abuse. Oral and written information was provided to each patient, and each patient had time to ask questions regarding the study. Ultimately, 125 patients provided their consent for inclusion in this study; however, due to surgery cancellations, 120 patients participated in this study (Figure 1).

### 2.2. Perioperative Care

Standardized endotracheal combined intravenous-volatile general anesthesia was administered, including premedication with oral paracetamol and intraoperative analgesia with a remifentanil infusion to maintain a Surgical Pleth Index (Carescape™ B650; GE Healthcare, Helsinki, Finland) between 20 and 50.

### 2.3. Postoperative Analgesia

For the first five postoperative days, the participants were randomly assigned to one of two groups using a randomization plan generator (www.randomization.com). In the active group, patients were provided tramadol/paracetamol (37.5 mg/325 mg tablets; two tablets; Trampalgin^®^, Weifa, Oslo, Norway). In the control group, two near-matched placebo tablets were administered preoperatively. After surgery, patients were advised to take two tablets twice a day for moderate to severe pain for a maximum of 5 days (a maximum of 20 tablets). To achieve standardized multimodal pain management in the hospital, patients were administered nonsteroidal anti-inflammatory analgesics: intravenous dexketoprofen and oral ibuprofen. Rescue analgesia was provided using intravenous or sublingual/peroral oxycodone when the patient reported pain at rest of at least three or dynamic pain of at least on an 11-point numeric rating scale (NRS; 0 = no pain, 10 = most pain). At discharge, each patient was provided with 30 ibuprofen tablets (200 mg; Burana^®^, Orion Corporation, Espoo, Finland) and was instructed to use up to 1200 mg/day as needed if they were unsatisfied with their pain relief from the study medicine. The attending surgeon prescribed additional analgesics to be used after the 5-day study period.

### 2.4. Data Collection

Four questionnaires were conducted before surgery at the hospital, completed by the patients themselves, and at 28 days and 12 months after surgery via phone call by one of the authors (EL, PT, or MK).

Pain severity and interference with daily activities were evaluated using a modified version of the Brief Pain Inventory (BPI) questionnaire [16,30]. The BPI assesses pain severity through four questions (average pain, least pain, and most pain during the previous 24 h, as well as pain right now) on an 11-point NRS. Pain interference was evaluated for eleven aspects on BPI: general activity, mood, walking ability, normal work (both work outside the home and housework), relations with other people, sleep, enjoyment of life, dressing up, lifting, sitting, and standing, on a scale of 0 (does not interfere) to 10 (completely interferes) [16,30]. The average score of the 11 items was used as the pain interference BPI score. The data were dichotomized, and scores of 0–4.9 indicated no or mild interference, while scores of 5–10 indicated moderate to severe interference [31]. Internal consistency with the current sample was moderately acceptable at both time points (Cronbach’s alpha = 0.508).

Life satisfaction was assessed using the Life Satisfaction Scale-4 (LS-4), which includes four items: interest in life, happiness, ease of living, and loneliness. The total score ranges from 4 to 20 and was classified into three groups: satisfied (LS-4 score of 4–6), intermediate (score of 7–11), and dissatisfied (score of 12–20) [32]. Internal consistency with the current sample was moderately acceptable at both time points (Cronbach’s alpha = 0.540).

Patients’ resilience was measured using the Resilience Scale-14 (RS-14). Each of the 14 items of this questionnaire is scored using a seven-point Likert scale (1 = strongly disagree, 7 = strongly agree), and the total scores range from 14 to 98, from very low resilience to high resilience. The resilience scores were dichotomized into low-resilience (14–70) and moderate or high-resilience (71–98) groups [33]. Internal consistency with the current sample was highly acceptable at both time points (Cronbach’s alpha = 0.799).

Anxiety and depressive symptoms were evaluated using the Hospital Anxiety and Depression Scale (HADS). The HADS contains 14 items, including seven questions evaluating anxiety symptoms and seven depression symptoms using a 0–3 scale of increasing anxiety or depression. The total score can range from 0 to 21 points for each dimension. The results were dichotomized into no or mild symptoms (0–10 points) and moderate to severe symptoms (11–21 points) for both anxiety and depression [34]. Internal consistency with the current sample was highly acceptable at both time points for anxiety (Cronbach’s alpha = 0.738) and for depression (Cronbach’s alpha = 0.697).

Patients completed the questionnaires (BPI, LS-4, RS-14, and HADS) preoperatively on the morning of the operation, and one of the authors was available for consultation. After discharge, the patients were interviewed by phone (EL, PT, or MK) at 28 days and 12 months after surgery to complete the follow-up questionnaires (Figure 1).

### 2.5. Outcome Measures

The primary outcome measures included life satisfaction, measured using the LS-4 scale, and pain interference, measured using the BPI at 12 months, as well as whether these two PROMs were associated with anxiety and depression symptoms and resilience. The secondary outcome measures were anxiety and depression symptoms, the resilience score, pain intensity, and interference, as well as life satisfaction in the placebo and tramadol–paracetamol groups at 12 months.

### 2.6. Statistics

The sample size calculation is based on the achievement of good or excellent pain relief achieved with tramadol–paracetamol (47%) and placebo (5%) in dental surgery patients with a statistical power of 90% [35]. The data were recorded and analyzed using Statistical Package for Social Sciences Software (IBM SPSS Statistics 27, International Business Machines Corporation, Armonk, NY, USA). The distribution of continuous data was visually checked, and the normal distribution assumption was tested using the Shapiro-Wilk test. Since the data were not normally distributed, continuous data were analyzed using the Wilcoxon Signed-Rank test for related variables and the Mann-Whitney U test for independent variables. Binomial and categorical variables were analyzed using the chi-square test and the McNemar test, as appropriate. The multivariate relationship between preoperative anxiety, depression, and resilience scores and life satisfaction and pain interference at 12 months after surgery were examined using a multivariate linear regression model. Continuous data are presented as the median and interquartile range (IQR), and categorical data are presented as numbers and percentages. *p*-values < 0.05 were considered statistically significant.

## 3. Results

### 3.1. Study Population

The patient characteristics are presented in Table 1. A total of 118 patients completed the questionnaires at 28 days, and 113 patients completed the questionnaires at 12 months postoperatively (94% response rate), including 56 patients in the placebo group and 57 patients in the tramadol–paracetamol group. There was no gender effect.

### 3.2. Primary Outcome Measures

The proportion of patients reporting satisfaction with their life increased significantly in both groups, in the placebo group, from 3 out of 59 patients (5%) before surgery to 23 out of 56 patients (41%) at 12 months after surgery (*p* < 0.001, the chi-square test) and in the tramadol–paracetamol group, from 2 out of 61 patients (3%) before surgery to 19 out of 57 patients (34%) at 12 months after surgery (*p* < 0.001, the chi-square test), respectively. The proportions of patients who were satisfied with their lives were similar for the two groups both at baseline (*p* = 0.459, the chi-square test) and at 12 months (*p* = 0.087, the chi-square test) (Table 2). The median of LS-4 score decreased (i.e., life satisfaction improved) from 9 [IQR, 9–11] to 7 [IQR, 6–7] (*p* < 0.001, d-value 0.890, Z-value −5.184, the Wilcoxon Signed-Rank test) in the placebo group and from 9 [IQR, 9–12] to 7 [IQR, 5–10] (*p* < 0.001, d-value 1.243, Z-value −4.824, the Wilcoxon Signed-Rank test) in the tramadol–paracetamol group, respectively. There was no difference between the groups at baseline (*p* = 0.419, Z-value −0.808, the Mann-Whitney U test) nor at 12 months (*p* = 0.396, Z-value −0.849, the Mann-Whitney U test) (Table 3).

Pain interference significantly decreased during the 12-month follow-up period. In the placebo group, the median decreased from 4.1 before surgery to 1.2 after surgery (*p* < 0.001, d-value 0.881, Z-score −4.148) at 12 months after surgery, and in the tramadol–paracetamol group, it decreased from 4.4 to 1.9 (*p* < 0.001, d-value 0.948, Z-score −5.151) at 12 months after surgery. There was no difference between the groups at baseline (*p* = 0.527, Z-value −0.633) nor at 12 months (*p* = 0.323, Z-value −0.885). The proportions of patients experiencing no or mild pain interference versus moderate or severe interference were also similar in both groups at baseline (*p* = 0.328) and at 12 months (*p* = 0.323) (Table 3).

The linear regression analysis did not show any statistically significant predictive value for preoperative anxiety, depression, or resilience score in relation to life satisfaction and pain interference at 12 months after spine surgery (Table 4).

### 3.3. Secondary Outcome Measures

The median [IQR] preoperative RS-14 score was higher in the placebo group (87 [79–93]) than in the tramadol–paracetamol group (83 [77–89]) (*p* = 0.045). Other psychological factors and pain scores were similar between the two groups. At 12 months postoperatively, all secondary outcome measures were similar between the groups and had significantly improved from the preoperative scores (Table 4).

Preoperatively, moderate to severe anxiety was present in four patients in the placebo group and nine in the tramadol–paracetamol group, while depression symptoms were noted in two and three patients, respectively. Both conditions were mostly alleviated by 12 months post surgery. Preoperatively, eleven patients (n = 2 and n = 9) reported low resilience compared with three patients (n = 1 and n = 2) at 12 months (Table 2).

Three of the thirteen patients with anxiety also had depression, and one had low resilience. One patient with anxiety, depression, and low resilience was dissatisfied with life and had substantial pain interference after surgery. One-third (4/13) of the patients with preoperative anxiety were dissatisfied with life and had substantial pain interference after surgery. One of the eleven patients with low preoperative resilience was dissatisfied with life, and two had substantial pain interference at 12 months after surgery.

**Figure 2:**
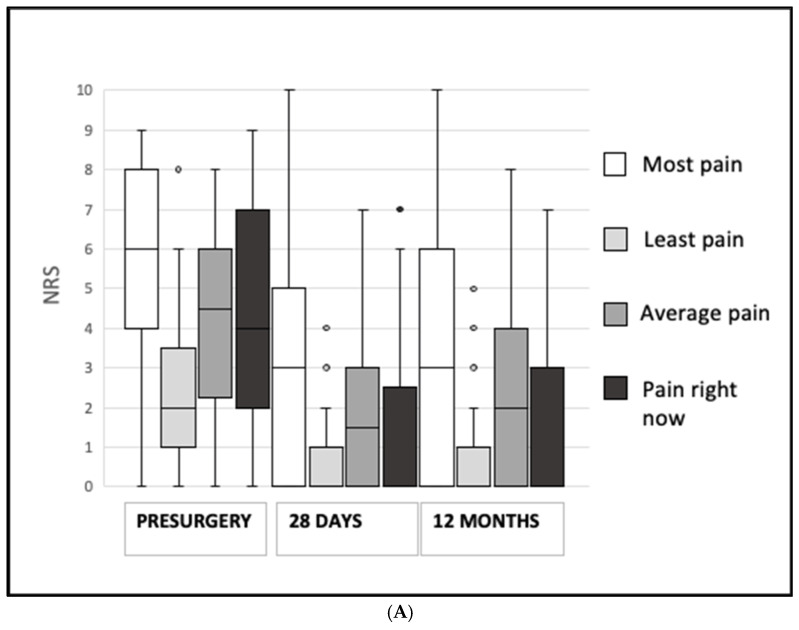

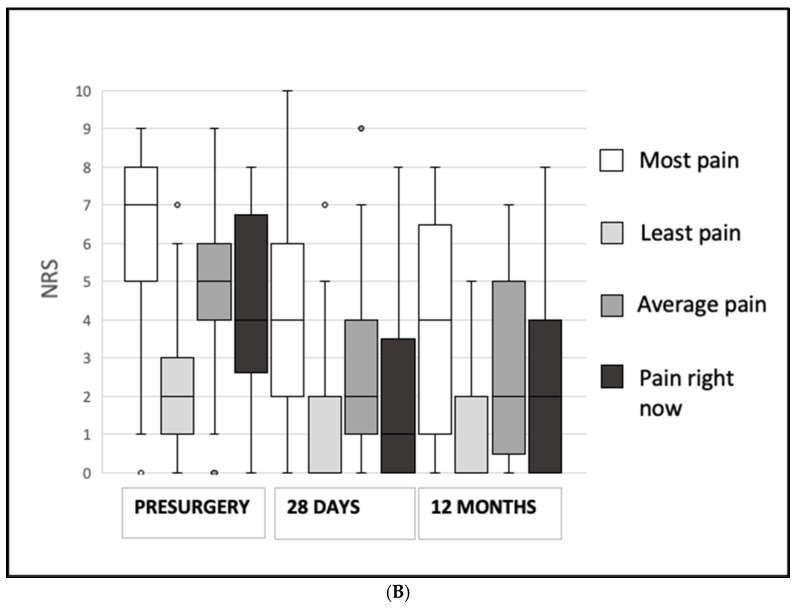
(**A**) The patients’ pain intensity before surgery and at 28 days and 12 months after surgery measured using a numeric rating scale (NRS 0–10) in the placebo group. The boxes represent quartiles with medians, the whiskers represent minimum and maximum values, and (O) represents outliers. (**B**) The patients’ pain intensity before surgery and at 28 days and 12 months after surgery measured using a numeric rating scale (NRS 0–10) in the tramadol–paracetamol group. The boxes represent quartiles with medians, the whiskers represent minimum and maximum values, and (O) represents outliers.

Similarly, the pain scores decreased significantly in both the placebo (*p* < 0.001) and the tramadol–paracetamol (*p* < 0.001) groups (Figure 2). Opioid analgesics were used before surgery by 15 (25%) patients in the placebo group, by 18 (30%) patients in the tramadol–paracetamol group (*p* = 0.616), and at 12 months postoperatively by 5 (9%) patients and 3 (5%) patients (*p* = 0.448) in the two groups, respectively. The decrease in opioid analgesic use was more substantial in the tramadol–paracetamol group (*p* < 0.001 baseline vs. 12 months) than in the placebo group (*p* = 0.020). In the placebo group, three of the five patients, and in the tramadol–paracetamol group, two of the three patients, were new opioid users. Twelve months after surgery, the prevalence of PSPS was lower in patients in the tramadol–paracetamol group, with 6 out of 56 (10%) patients reporting pain exacerbation compared with presurgery pain. In comparison, 13 out of 57 (22%) patients in the placebo group reported worsening pain (*p* = 0.086). One patient in each group reported radicular symptoms that began after surgery.

## 4. Discussion

In this study, the use of tramadol–paracetamol as an add-on pain treatment for the first five postoperative days after spine surgery did not affect patient-reported outcome measures (PROMs), life satisfaction, or pain interference at 12 months after surgery. Mental health symptoms of anxiety, depression, and low resilience also had no effect on these outcome measures. This contradicts the findings presented by Epker and Block [36] in their review two decades ago, as well as more recent studies by Skeppholm et al. [37] and Tuomainen et al. [38]. Previous studies have suggested that preoperative anxiety and depression symptoms are associated with worse long-term outcomes, higher pain scores, and increased disability for up to 10 years after spine surgery.

The novelty of this study lies in evaluating the predictive value of resilience on patient life satisfaction and pain interference 12 months after spine surgery. In terms of surgical patient outcomes, resilience has received less research attention than the other two psychological factors, anxiety and depression [39]. Resilience means a patient’s capacity to adapt in the face of adversity [11]. The mechanism of resilience is not fully understood, but it is thought to be an emotional and cognitive process to avoid negative effects during the adaptation process through unexpected situations that could cause emotional distress and pain [40]. Contrary to our data, other studies have found resilience to be an important predictive psychosocial factor for some PROMs. For instance, Daher and colleagues recently reported that preoperative resilience is associated with patients’ pain, physical performance, health-related quality of life, and physical and mental health 12 months after spinal fusion surgery [13]. Coronado et al. measured resilience 6 weeks after spine surgery and reported correlations with disability, back pain, pain interference, physical function, and social participation 12 months after surgery [41]. In a *post hoc* analysis of our data, it was found that resilience scores at 4 weeks after surgery had no predictive value for life satisfaction and pain interference at 12 months after surgery.

Preoperative screening has been proposed, as resilience is a psychological factor that can be improved [12]. Adogwa and colleagues reported that identifying and treating psychiatric symptoms before spine surgery improves clinical outcomes. Patients with anxiety and depression who receive appropriate preoperative treatment report better postoperative outcomes compared with those who do not receive treatment beforehand. In Adogwa and colleagues’ studies, patients with known preoperative anxiety or depression who received appropriate treatment had long-term outcomes similar to those of patients without preoperative psychological symptoms [42,43].

In the current study, most patients reported relatively good life satisfaction and low pain interference postoperatively. This study also revealed that anxiety and depression symptoms were alleviated in most patients, and a high resilience score was more common at 12 months after surgery than before surgery. These data are consistent with previous reports from our institution [16,38,44] and highlight the potential benefits of successful patient selection for spine surgery on overall physical, psychological, and social health. In the present study, 20% of patients were using opioid analgesics before surgery, which decreased to just 7% after surgery, with a more substantial decrease in the tramadol–paracetamol group. Six out of eleven patients with low resilience before surgery were also using preoperative opioid analgesics, but only one of those patients used opioids at 12 months. At 12 months, three low-resilience patients did not require opioid analgesics.

The findings of the current study are strengthened by the long 12-month follow-up period and the use of validated questionnaires completed by the patients before surgery, at 4 weeks, and at 12 months after surgery. Trained researchers collected the data and guided the participants to ensure their understanding of the questions.

Limitations of this study include a relatively small sample size of 120 patients—with few subjects showing significant symptoms of anxiety, depression, or low resilience. However, the response rate was high (>98% at four weeks and >94% at 12 months). The exclusion criterion related to BMI is also questionable, particularly given that anxiety and depression are multifactorial and may be influenced by a range of physical and psychological factors. Additionally, some patients may have reported pain outcomes 12 months after surgery unrelated to their spine condition, but the use of the Brief Pain Inventory and body diagrams minimized confusing spinal pain with other conditions [30]. Moreover, PROMs may be influenced by internal factors, like mood and expectations, but the randomized placebo-controlled double-blinded clinical trial, the evaluation of PROMs at multiple time points, and the long follow-up suggest minimal bias.

Further studies are needed to determine if postoperative outcomes can be improved by supporting and training resilience skills and treating psychiatric symptoms before spine surgery to optimize surgical timing.

## 5. Conclusions

These results highlight that the use of tramadol–paracetamol for add-on pain treatment, as well as psychological factors, anxiety, depression, and resilience, did not affect PROMs, postoperative pain interference, or life satisfaction in patients undergoing spine surgery.

## Figures and Tables

**Table 1:** Patient characteristics. Data are presented as the number of cases (%) or median [interquartile ranges, IQRs].

Parameter	All Patients, n = 120	Placebo Group, n = 59	Tramadol–Paracetamol Group, n = 61
Sex, male/female, n (%)	74 (62%)/46 (38%)	37 (63%)/22 (37%)	37 (61%)/24 (39%)
Age, years, median [IQR]	54 [48–61]	58 [52–64]	52 [45–58]
Weight, kg, median [IQR]	82 [74–92]	82 [73–94]	83 [75–92]
Height, cm, [IQR]	174 [168–181]	175 [167–181]	174 [168–180]
Lumbar/Cervical surgery, n (%)	59 (49%)/61 (51%)	28 (47%)/31 (53%)	31 (51%)/30 (49%)

**Table 2:** Survey scores at baseline and 28 days and twelve months after surgery. Data are presented as number of cases (%).

Variable	Life Satisfaction (LS-4) Scale 4–20	Pain Interference (BPI) Scale 0–10	Anxiety (HADS) Scale 0–21	Depression (HADS) Scale 0–21	Resilience (RS-14) Scale 14–98
Baseline					
Classes ^1^ (n (%)) Placebo (n = 59)	3 (5%)/44 (76%)/11 (19%)	17 (30%)/42 (70%)	4 (7%)/55 (93%)	2 (3%)/57 (97%)	2 (4%)/54 (96%)
Tramadol–paracetamol (n = 61)	2 (3%)/41 (69%)/17 (28%)	22 (37%)/37 (63%)	9 (15%)/50 (85%)	3 (5%)/55 (95%)	9 (16%)/46 (84%)
28 days					
Classes ^1^ (n (%)) Placebo (n = 57)	22 (39%)/35 (61%)/0 (0%)	1 (2%)/56 (98%)	1 (2%)/55 (98%)	0 (0%)/57 (100%)	1 (2%)/56 (98%)
Tramadol–paracetamol (n = 61)	25 (41%)/28 (46%)/8 (13%)	9 (15%)/52 (85%)	9 (15%)/50 (85%)	3 (5%)/55 (95%)	8 (13%)/53 (87%)
12 months					
Classes ^1^ (n (%)) Placebo (n = 56)	23 (41%)/31 (55%)/2 (4%)	7 (12%)/49 (88%)	0 (0%)/56 100%)	0 (0%)/56 (100%)	1 (2%)/53 (98%)
Tramadol–paracetamol (n = 57)	19 (34%)/29 (50%)/9 (16%)	11 (19%)/46 (81%)	2 (4%)/55 (96%)	1 (2%)/56 (98%)	2 (4%)/53 (96%)
Baseline—12 months					
Placebo					
*p*-value	<0.001	0.031	0.119	0.496	1.0
Tramadol–paracetamol					
*p*-value	<0.001	0.032	0.031	0.618	0.069

HADS, Hospital Anxiety and Depression Scale; ^1^ Classes: Life Satisfaction-4: satisfied 4–6, intermediate 7–11, dissatisfied 12–20; Pain Interference: 0–4.9/5.0–10; Anxiety: 0–10/11–21; Depression: 0–10/11–21; Resilience-14: 14–70/71–98.

**Table 3:** Anxiety, depression, resilience, pain intensity, pain interference, and life satisfaction before surgery and 12 months after surgery in the tramadol–paracetamol and placebo groups. Data are presented as medians [interquartile ranges, IQRs] and *p*-values between the groups.

Parameter		Placebo Group n = 56	Tramadol–Paracetamol Group	*p*-Value
Anxiety: Hospital Anxiety and Depression Scale 0–21, median [IQR]				
Before surgery		5 [4–8]	6 [4–8]	0.555
At 12 months		4 [2–7]	5 [3–7]	0.196
Depression: Hospital Anxiety and Depression Scale 0–21, median [IQR]				
Before surgery		3 [2–6]	4 [2–7]	0.255
At 12 months		2 [0–4]	2 [0–5]	0.784
Resilience: Resilience Scale-14 14–98, median [IQR]				
Before surgery		87 [79–93]	83 [77–89]	0.045
At 12 months		89 [83–95]	87 [80–94]	0.436
Pain intensity: Brief Pain Inventory 0–10, median [IQR]				
Most pain	Before surgery	6 [4–8]	7 [5–8]	0.325
	At 12 months	3 [0–6]	4 [1–7]	0.483
Least pain	Before surgery	2 [1–4]	2 [1–3]	0.914
	At 12 months	0 [0–1]	0 [0–2]	0.376
Average pain	Before surgery	5 [2–6]	5 [4–6]	0.208
	At 12 months	2 [0–4]	2 [1–5]	0.452
Pain right now	Before surgery	4 [2–7]	4 [3–7]	0.609
	At 12 months	0 [0–3]	2 [0–4]	0.356
Pain Interference: Brief Pain Inventory 0–10, median [IQR]				
Before surgery		4.1 [2.5–5.9]	4.4 [2.3–6.3]	0.395
At 12 months		1.2 [0–4.0]	1.9 [0.0–4.6]	0.376
Life Satisfaction: Life Satisfaction (LS-4) 4–20, median [IQR]				
Before surgery		9 [9–11]	9 [9–12]	0.419
At 12 months		7 [6–7]	7 [5–10]	0.396

**Table 4:** The multivariate linear regression analysis coefficients beta values between anxiety, depression, and resilience before surgery and life satisfaction and pain interference 12 months after spine surgery.

Variable	Anxiety (HADS)	Depression (HADS)	Resilience (RS-14)
Unstandardized/Standardized Coefficient B values			
Life satisfaction (LS-4) at 12 months			
Placebo group	−0.100/−0.114 ^a^	0.076/0.100 ^a^	0.087/0.296 ^a^
*p*-value	0.480	0.582	0.069
Tramadol–paracetamol group *p*-value	0.210/0.240 ^a^ 0.178	0.247/0.244 ^a^ 0.128	0.051/0.189 ^a^ 0.231
Pain interference (BPI) at 12 months			
Placebo group	−0.110/−0.137 ^b^	0.227/0.327 ^b^	0.012/0.046 ^b^
*p*-value	0.400	0.080	0.777
Tramadol–paracetamol group *p*-value	0.153/0.231 ^b^ 0.224	0.218/0.287 ^b^ 0.097	−0.004/−0.019 ^b^ 0.910

HADS, Hospital Anxiety and Depression Scale; ^a^ standardized to Life satisfaction, ^b^ standardized to Pain interference.

## Data Availability

The raw data can be obtained upon request from the corresponding author.
